# Food Colors’ Dietary Exposure in the Brazilian Population Using the 2008–2009 and 2017–2018 POF Food Consumption Databases

**DOI:** 10.3390/foods13244006

**Published:** 2024-12-11

**Authors:** Larissa Bertollo Gomes Pôrto, Adriana Pavesi Arisseto Bragotto

**Affiliations:** 1 Faculdade de Engenharia de Alimentos, Universidade Estadual de Campinas, Cidade Universitária, R. Monteiro Lobato 80, Campinas 13083-862, Brazil; larissa.porto@anvisa.gov.br; 2 Brazilian Health Regulatory Agency (ANVISA), SIA Trecho 5 Área Especial 57, Brasília 71205-050, Brazil

**Keywords:** risk assessment, ingestion, food additives, color, food safety

## Abstract

Two out of the four steps of risk assessment for chemical substances in food, i.e., exposure assessment and risk characterization, merit regional evaluation based on current legislation and local food consumption data. Therefore, mean and high exposures to food colors were estimated in Brazil using a conservative approach to screen substances with a higher risk of the exceedance of safety parameters. Brazilian National Consumption Surveys from the Household Budget Surveys (POF—Pesquisa de Orçamentos Familiares) from 2008–2009 and 2017–2018 were combined with the maximum permitted levels of 33 food colors. Higher exposure estimates were obtained for the oldest POF database. High priority for a refined exposure assessment was identified for six food colors for which the mean and high exposures were higher than the safety parameters, while medium priority was observed for eleven food colors for which the mean exposures were below but the high exposures were above the safety parameters. Low priority was noted for 16 substances for which no exceedance was obtained despite the conservativeness of the methodology applied. The prioritization of food colors for future risk assessments was achieved to identify substances for which more refined exposure methodologies are necessary to characterize the risk to health.

## 1. Introduction

To be permitted for use in food, substances such as food additives, which are not normally consumed as food but perform a technological function, must be considered safe [1,2]. For this, one of the conditions required is that the intake of the food additive be below the acceptable daily intake (ADI), a safety parameter which corresponds to the amount that can be “ingested daily over a lifetime without appreciable health risk” [1,3]. The estimation of the intake of a chemical substance by a population is known as exposure assessment, which is one of the four steps of the scientifically based process called risk assessment used to assess possible risks due to chemical intake [4].

Numerous methodologies can be used to evaluate exposure as described by the International Programme on Chemical Safety (IPCS) [5]. As a screening method, the IPCS mentions the use of maximum levels (MLs) allowed for food additives, which leads to an intentional overestimation that also protects individuals considered “high consumers” [5]. This term is used to define individuals that may be exposed to high amounts of a substance under consideration either by consuming high amounts of food containing the chemical or by consuming several foods, in regular amounts, that have the same substance [5,6]. MLs are combined with consumption data, preferably using mean individual data from surveys of at least two non-consecutive days [5,7,8].

Exposure assessments of food colors’ consumption by the Brazilian population are scarce and focus only on a few substances. Consumption data from the National Consumption Surveys within the scope of the Household Budget Survey (Pesquisa de Orçamentos Familares—POF; for the Portuguese acronym) from 2008–2009 and 2017–2018 from the National Institute of Geography and Statistics (Instituto Brasileiro de Geografia e Estatística—IBGE; for the Portuguese acronym) were used to evaluate exposure to INS 102 tartrazine, INS 110 sunset yellow FCF, INS 129 Allura red AC, INS 124 ponceau 4R, INS 127 erythrosine, INS 123 amaranth, INS 122 azorubine, INS 132 indigotin, and INS 133 brilliant blue FCF [9,10,11,12,13]. To our knowledge, exposure assessment for other food colors with established numerical ADIs has been performed only considering the Study of Cardiovascular Risks in Adolescents (Estudo de Riscos Cardiovasculares em Adolescentes—ERICA; for the Portuguese acronym) food consumption database [8].

This study aimed to prioritize food colors allowed in Brazil using food consumption data from the POF from 2008–2009 and 2017–2018 as a screening methodology to enable the identification of substances for which more robust evaluations may be needed using more detailed data. For that, exposure estimates at mean and high levels were calculated and were compared, respectively, to the ADIs set by the Joint FAO/WHO Expert Committee on Food Additives (JECFA), using as concentration data the MLs from Brazilian legislation [2,14,15].

## 2. Materials and Methods

### 2.1. Food Colors

Food colors considered in the present assessment were previously identified as described by [8]. A total of 35 colors out of the 62 permitted in Brazil were evaluated by the JECFA with a numerical ADI, but exposure estimates were performed here for 33 due to the following reasons: (i) no consumption data for the enteral nutrition formula were available, which is the only category allowing INS 160a(iv) beta-carotene-rich extract from *Dunaliela salina*, and (ii) no concentration data were available for INS 173 aluminum powder in sugar- and chocolate-based toppings for baked goods, edible ices and confectionery, which are the categories for which the substance is allowed in Brazil.

### 2.2. Consumption Data

Brazilian National Consumption Surveys from the POF from 2008–2009 and 2017–2018 were used as food consumption data, which were acquired on two non-consecutive days covering residents from 10 years old to older living in selected residences. In the 2008–2009 survey, food consumption data were collected through food records and covered 3569 residences, representing 24.3% of 55,970 residences included in the original survey, corresponding to a population of 34,003 individuals [16]. In the 2017–2018 survey, a subset of 34.7% of 57,920 residences was selected, comprising 20,112 residences, and included food consumption data from 46,164 participants using a 24 h recall method [17]. Detailed information on the surveys is available in the literature [16,17].

Raw food consumption data were extracted [18,19,20] from the IBGE website and mean and high percentile (P90 and P95) values were calculated in grams per body weight considering a chronic assessment and using expansion factors as released by the IBGE [18,19,20], grouping food into categories based on the Brazilian legislation [14,15]. The literature was used to estimate ingredient percentages in the case of food preparations [21,22,23], using available online recipes (https://www.cozinhanet.com.br, (accessed on 14 September 2023)) as an additional source when necessary. The consumption data were then firstly calculated individually by using individual body weights and dividing by two (number of days), and then mean values of food consumption were obtained for the whole population studied (population) while high percentiles of food consumption (P90 and P95) were calculated considering only individuals who reported the consumption of the specific food (consumers only) from each category.

### 2.3. Exposure Estimates

Exposure estimates were performed using the same methodology as described by [8], obtaining mean and high estimates. Briefly, mean estimates were calculated considering the mean food consumption of the whole population investigated in each survey and maximum levels of food colors allowed for each food category. The estimates obtained for each food category were then summed to obtain the total exposure for individual food colors. The percentage (%) of contribution of each food category to the total exposure of the color was calculated as follows: (estimated intake of the food category × 100)/total estimated intake. To obtain the exposure for high consumers, i.e., consumers that may be exposed to high levels of the substances, estimates were conducted by replacing the mean consumption data for the food category that most contributed to the mean estimate by food consumption at P90 and P95 (calculated for consumers only), whereas mean food consumption values (calculated for the whole population) were used for the remaining food categories. MLs were considered as concentration data in all scenarios.

### 2.4. Food Colors Prioritization

Dietary exposures were compared with the ADI values for each food color, and the percentage of ADI exceedance was used to classify colors as low, medium, or high priority for a refined exposure assessment. Low priority was designated for substances for which all estimates were lower than the ADI; medium priority was considered when one of the estimates was higher than the ADI; and high priority was considered when all obtained estimates were above the ADI.

## 3. Results

### 3.1. Exposure Assessment

Mean dietary exposure estimates for food colors’ intake by the Brazilian population are shown in Table 1. Estimates above the ADI using both databases were obtained for INS 150d caramel IV (sulfite ammonia caramel), INS 160a(i) synthetic beta-carotenes, INS 160a(iii) beta-carotenes from *Blakeslea trispora*, INS 160e beta-apo-8′-carotenal, and INS 163(iii) blackcurrant extract. Besides these colors, INS 150b caramel II (sulfite caramel) exceeded the ADI only when the oldest survey (2007–2008) was considered. One possible explanation for the high values obtained for these colors is the fact that they are overall authorized in many food categories and that the methodology used may have highly overestimated the estimates.

In general, higher estimates were obtained using the POF from 2008–2009 compared to the 2017–2018 survey, which were related to differences in the consumption data as the concentration values used were the same ones. A similar pattern was verified by [10,12,13], who observed a reduction in exposure estimates using 2017–2018 POF data for INS 110 sunset yellow FCF, INS 123 amaranth, and four colors (INS 122 azorubine, INS 127 erythrosine, INS 132 indigotin, and INS 124 ponceau 4R), respectively, compared to the POF from 2008–2009. The largest decreases were observed for INS 133 brilliant blue FCF, INS 172(i), (ii), (iii) iron oxides (black, red, and yellow), INS 173 aluminum powder, and INS 183 jagua (genipin–glycine) blue, with a reduction of at least 50%. It is worth mentioning that INS 183 was only approved in Brazil in 2023, so the population was not exposed to this ingredient when both POFs were undertaken.

According to Table 2, estimates for high consumers using P90 of food consumption (consumers only) for the food that most contributed to the mean exposures were at or above the ADI for 14 colors considering both surveys: INS 123 amaranth, INS 127 erythrosine, INS 150b caramel II (sulfite caramel), INS 150c caramel III (ammonia caramel), INS 150d caramel IV (sulfite ammonia caramel), INS 160a(i) synthetic beta-carotenes, INS 160a(iii) beta-carotenes from *Blakeslea trispora*, INS 160b(ii) annatto extracts (bixin-based), INS 160c(ii) paprika extract, INS 160e beta-apo-8′-carotenal, INS 163(iii) blackcurrant extract, and INS 172(i), (ii), (iii) iron oxides. The INS 124 Ponceau 4R estimate for high consumers using P90 was slightly above the ADI with the oldest POF but was half of the ADI with the newest survey. Similar results were also seen when P95 was used, apart from for INS 151 brilliant black, with exposures above the ADI at P95 but below the safety parameter at P90 for both surveys, and INS 161g canthaxanthin, which showed exposures higher than the ADI for the POF from 2008–2009 and below the ADI for the POF from 2017–2018.

The reductions in the estimates seen in the mean exposures (Table 1) were also observed in the high-exposure scenario using P90 (Table 2), except for INS 104 quinoline yellow and INS 151 brilliant black, for which estimates were higher with the POF from 2017–2018. In the P95 scenario (Table 2), lower estimates were also noted when using the POF from 2017–2018 for most of the substances evaluated, but they were greater in nine cases: INS 102 tartrazine, INS 104 quinoline yellow, INS 122 azorubine, INS 123 amaranth, INS 127 erythrosine, INS 150b caramel II (sulfite caramel), INS 150d caramel IV (sulfite ammonia caramel), INS 151 brilliant black, and INS 155 brown HT.

### 3.2. Main Food Categories in Exposure Assessment

Largely, “water-based flavored drinks” was the main food category that impacted the mean exposure to food colors, followed by “fish and fish products” and “pasta and noodles” (Table 3). Generally, the main contributor was the same category in both surveys for a specific color, except for INS 110, INS 129, INS 132, INS 133, INS 160a(i), INS 160b(i), INS 160b(ii), and INS 161g. As also noted by [8], the most consumed food was not always the greatest source of exposure.

Overall, for the exposures estimates in which “water-based flavored beverages” were the main contributor (corresponding to at least 40% of the estimates), higher values were calculated in the scenario for high consumers using the most recent survey as compared to the older one. However, some food colors also had “water-based flavored beverages” as an important contributor to the exposures, varying from 35 to 56% of the estimates, but lower estimates were obtained for the POF from 2017–2018. This was so in the following cases: INS 143 fast green FCF; INS 110 sunset yellow FCF; INS 129 Allura red AC; INS 132 indigotin; INS 133 brilliant blue FCF; INS 141(i) chlorophylls, copper complexes; and INS 141(ii) chlorophyllin copper complexes, potassium, and sodium salts.

For INS 132 indigotin, INS 129 Allura red AC, INS 133 brilliant blue FCF, and INS 110 sunset yellow, “fish and fish products” was the category that contributed most to the mean exposure using the POF from 2008–2009, while “water-based flavored drinks” had a higher influence when using the POF from 2017–2018. A reduction in the consumption of “fish and fish products” was observed between the two surveys, with a mean consumption (total population) of 0.6 g kg BW^−1^ day^−1^ for the POF from 2008–2009 and of 0.2 g kg BW^−1^ day^−1^ for the POF from 2017–2018 and a P90 consumption of 8 g kg BW^−1^ day^−1^ for the POF from 2008–2009 and of 3.6 g kg BW^−1^ day^−1^ for the POF from 2017–2018. This reduction may have balanced the higher consumption of “water-based beverages” and could explain the overall reduction in exposure for some colors. A decrease in the mean consumption was also noted for juices and pastas that, together with the reduction observed for “water-based flavored beverages” and for “fish and fish products”, contributed to the lower estimates for INS 141(i) chlorophylls, copper complexes.

### 3.3. Food Colors Prioritization

ADIs were not exceeded in any case for the following: INS 100(i) curcumin; INS 120 carmines; INS 102 tartrazine; INS 104 quinoline yellow; INS 110 sunset yellow FCF; INS 122 azorubine; INS 129 Allura red AC; INS 132 indigotin; INS 133 brilliant blue FCF; INS 141(i) chlorophyll copper complexes; INS 141(ii) chlorophyllin copper complexes, potassium, and sodium salts; INS 143 fast green FCF; INS 155 brown HT; INS 160b(i) annatto extracts (bixin-based); INS 163(ii) grape skin extract; and INS 183 jagua (genipin–glycine) blue. The mean and high exposure (P90) estimates were lower than the ADIs for the following: INS 123 amaranth; INS 127 erythrosine; INS 133 brilliant black; and INS 161g canthaxanthin. However, high exposures using P95 were slightly higher than the respective ADIs for these additives. Considering the 2008–2009 survey, the mean estimates for INS 124 ponceau 4R were lower than the safety parameter, but ADI exceedance was verified using high-exposures scenarios. Nevertheless, using the 2017–2018 data, the estimates were lower than the ADI in all scenarios for this color. Exposure estimates for the iron oxides (INS 172(i), 172(ii), and 172(iii)), INS 150c caramel III, INS 160c(ii) paprika extract, and INS 160b(ii) annatto extract (norbixin-based), were lower than the ADI for the mean- and high-exposure scenarios (P90 and P95). For INS 150b caramel II, INS 150d caramel IV, INS 160e beta-apo-8′-carotenal, INS 160a(i) synthetic beta-carotenes, INS 163(iii) blackcurrant extract, and INS 160a(iii) beta-carotenes from *Blakeslea trispora*, all estimates were higher than the ADI, except for INS 150b for the mean for the 2017–2018 survey, for which the estimate was close to the ADI. The classification of the food colors as low, medium or high priority for a refined exposure assessment based on the percentage of ADI exceedance is shown in Table 4.

## 4. Discussion

Although color is considered an important attribute of food, closely related to its quality and acceptance, it is imperative that the use of food colors does not pose risks to consumers. For this purpose, exposure assessment is an essential tool to verify whether the intake of food colors is below established safety parameters.

In Brazil, using the same food consumption databases, Ref. [11] also obtained mean estimates below the ADI for INS 102 tartrazine, INS 110 sunset yellow FCF, INS 122 azorubine, INS 129 Allura red AC, INS 132 indigotin, INS 133 brilliant blue FCF, INS 123 amaranth, INS 124 ponceau 4R, and INS 127 erythrosine. In the high-exposure scenario, the ADIs were exceeded for INS 102 tartrazine, INS 110 sunset yellow FCF, and INS 123 amaranth, while in this study the cited estimates were higher than the ADI for INS 123 amaranth, INS 124 ponceau 4R, and INS 127 erythrosine. Exposure estimates for INS 102 tartrazine calculated by [42], stratified by gender, region, per capita family income, or age (adolescents: 10–18 y; adults: 18–65 y; elderly > 65 y), were all below the ADI, in line with the results of the present study with no ADI exceedance. On the other hand, using the prevalence of food consumption to estimate high exposures, Ref. [42] verified ADI exceedance, especially for women (lower incomes), adolescents (both genders), and women up to 24 years old, whereas no estimate was above 20% of the ADI for INS 102 tartrazine in the present study even if only P95 of food consumption from consumers was considered. One possible explanation is that in their study [42], the prevalence was considered for all food categories for which the additive was found in at least one product, while the current study considered data for consumers only for a single food category, according to the methodology indicated by the IPCS [5].

Despite using a different methodology and applying stratifications, Ref. [9] also obtained estimates below the ADI for INS 110 sunset yellow FCF at mean and high exposures. However, when the authors considered the prevalence of food consumption for all food categories, the ADI was exceeded, but the resulting estimate was probably highly overestimated and may not have represented a real high exposure as an individual would not consume all food products in high amounts every day. In [9], the main food categories that contributed to the mean estimates were “fruit juices and nectars” and “water-based flavored drinks”, corresponding to almost 75%, while in the present study, “fish and fish products” and “water-based flavored drinks” were the main contributors to exposure, corresponding to 73% of the estimate. Besides the methodological difference, it is worth pointing that the Brazilian legislation has changed in the past years and provisions for food colors for “fish and fish products” were only officially listed in 2019 and further included in the legislation in 2023 [14,43].

Considering a more refined methodology that used raw individual data from the POFs [10], estimates were lower than the ones obtained by [9] and the ones calculated in the present study for INS 110 sunset yellow FCF. In [10], a high percentage of the investigated population in the surveys did not consume any food for which the color is allowed, with 86.62% in the POF from 2008–2009 and 92.57% in the POF from 2017–2018. For those that consumed foods containing INS 110 sunset yellow FCF, most estimates calculated by [10] were below the ADI, although the higher exposures were above the ADI of 4 mg kg BW^−1^ day^−1^.

Globally, the exposure assessment of food colors has been mainly conducted for artificial substances, especially azo dyes. According to a comprehensive review on the risk assessment of azo dyes as food additives [44], studies on exposure estimation have shown that the human oral intake of azo dyes is usually below the ADI. This was also observed in the present study, since artificial colors were classified as low and medium priorities for a refined risk assessment. However, Ref. [44] emphasized that most countries do not have studies that estimate the oral intake of azo dyes.

Due to different food additive provisions across different countries, direct comparisons with international exposure estimates are of limited value. As stated by [45], while international guidelines are widely recognized, they are not always incorporated into national and regional regulatory procedures, frameworks, and standards elaborated to ensure food safety. Despite global efforts to promote cooperation and harmonization, regulations still vary from one country to another. In 2017, for example, 39 colors were authorized as food additives in the EU while the US had 36 approved substances [45].

The Brazilian legislation [46] requires that food additives are declared on food labels at the end of the list of ingredients, either by the declaration of the name of the additive or by its International Number System (INS) number. Currently, there is no obligation to declare additives according to their relative proportion in food, but they must be grouped by technological function. Thus, if more than one color is used in a given food, they can be declared as “colors:”, followed by the names or INS numbers.

To our knowledge, two recent publications evaluated food labels in Brazil to verify the distribution and frequency of the use of food additives declared in commercial products. In one of them [47], 27.8% of the additives declared on the labels were related to colors. In the other one [48], it was observed that colors were present in almost all food groups, except “leafy vegetables” and “vegetables”, but only 29 out of the 62 permitted colors in Brazil were found in the evaluated products. Considering these 29 food colors, 22 had their exposure estimated in the present study. Annatto extracts, identified here as a medium priority for further evaluation, were the most used color with a numerical ADI, with 249 labels declaring the additive [48]. Two other colors were also frequently used according to [48]: carmines (identified here as a low priority, with 239 labels) and caramel IV (identified here as a high priority, with 218 labels).

Given the lack of quantitative information about food colors in the labels, one possibility to further evaluate the risk related to the possible extrapolation of the ADI values of those colors identified as a medium or high priority would be to first consider the individual data of food consumption. Another way forward would be to analytically identify the concentrations of colors in foods identified as the main contributors to exposure. Finally, the possibility of a call for data on their use levels could be considered by the regulatory authority.

### Limitations and Uncertainties

Uncertain sources must be pointed out in the present study, and one of them is related to the form of MLs’ expression, as for some food categories the legislation expresses the ML on a specific basis that may not be exactly how the ADI is expressed. For instance, grape and blackcurrant extracts are approved without any mention of the form of expression, but the ADI for anthocyanins and MLs in the General Standard for Food Additives (GSFA) are both expressed as “anthocyanins” bases. Additionally, the content of anthocyanins in ingredients may not be present in the specifications [38]. In the case of grape skin extracts, the ML was considered a content of 3% of anthocyanins, as described by the JECFA [27], but no information for blackcurrant was available and its exposures were compared to the ADI directly without any conversion, which may have overestimated the results. Another example is annatto extracts, which can contain at least 25% to 85% of bixin or at least 15% to 85% of norbixin, depending on the process used [27,38]. Considering the conservative approach used in this study, it was assumed that extracts obtained from annatto consisted mostly of pigments.

Due to limitations in the food consumption database, the contribution of some food categories to the exposure were not included, which could have underestimated the results. In addition, some food categories were not included simply because consumption data were not available. Also, some categories needed to be grouped as the databases do not differentiate products in the same way as the legislation. One example is that “fish and fish products” in the legislation are classified as being fresh (few additives permitted), industrialized, not heat-treated, cooked, fried, dried, or in brine, but available consumption data only define fish products’ consumption as a group. This may overestimate exposure as the color may not be allowed in all categories or at the same level, and the highest one was assumed in the present assessment.

Another source of overestimation is the assumption that every product within a category is made with the substance. In fact, the legislation may set a restriction on the use of colors for specific foods and not all products in the market may contain the substance. Also, even if the color is allowed, industries may not use it or may use another allowed color. Moreover, as already mentioned, the use of MLs overestimated the evaluation.

In cases where colors are allowed as *quantum satis*, the consideration that the level used would be equal to GSFA MLs could both under- or overestimate exposure as the level of use could be lower or higher. For some colors, the ADI is established on a specific basis, such as for INS 163(ii) and INS 163(iii), for which the ADI is expressed as an “anthocyanins base”. Nevertheless, MLs are not always expressed on the same basis, which can overestimate exposure.

Overall, the resulting estimations can be considered overestimated, and in cases where they were above the ADI, more refined methodologies of exposure assessment are required. For example, individual exposures should be estimated instead of aggregated food consumption data, and levels of use in food can be assumed as occurrence data instead of MLs.

Despite the uncertainties and limitations pointed out here, our findings are valid and the main positive aspects of this evaluation include the study of all food colors with numerical ADIs approved for use in Brazil, which, to our knowledge, is the first evaluation using representative consumption data for the country for the population aged 10 years and older. It is expected that the results obtained can contribute to the advancement of knowledge about the safety of these food additives and to the definition of regulatory priorities aimed at protecting consumer health.

## 5. Conclusions

Dietary exposure estimates for all colors allowed in Brazil that have a numerical ADI (or a safety parameter) were obtained using the national food consumption surveys from the POF from 2008–2009 and POF from 2017–2018, based on conservative assumptions. In general, a reduction in the exposure using the most recent database was observed. INS 100(i) curcumin, INS 120 carmines, INS 102 tartrazine, INS 104 quinoline yellow, INS 110 sunset yellow FCF, INS 122 azorubine, INS 129 Allura red AC, INS 321 indigotin, INS 133 brilliant blue FCF, INS 141(i) chlorophylls (copper complexes), INS 141(ii) chlorophyllin (copper complexes, potassium, and sodium salts), INS 143 fast green FCF, INS 155 brown HT, INS 160b(i) annatto extracts (bixin-based), INS 163(ii) grape skin extract, and INS 183 jagua (genipin–glycine) blue estimates were all below the respective ADIs and were thus considered low priorities for a refined exposure assessment. For INS 123 amaranth, INS 127 erythrosine, INS 151 brilliant black, INS 161g canthaxanthin, INS 124 ponceau 4R, INS 172(i, ii, iii) iron oxides (black, red, and yellow), INS 160b(ii) annatto extracts (norbixin-based), INS 160c(ii) paprika extract, and INS 150c caramel III, exposures could be higher than the ADI for high consumers. In the case of caramels II and IV (INS 150b and 150d, respectively), INS 160e beta-apo-8′-carotenal, INS 163(iii) blackcurrant extract, INS 160a(i) synthetic beta-carotenes, and INS 160a(iv) beta-carotenes from *B. trispora*, mean and high exposures were above the ADI, indicating a high priority for future risk assessments. Considering that the data obtained were probably highly overestimated in relation to the real exposures, our study corresponds to a prioritization approach for the identification of food colors for which exposure estimates could be higher than the ADI and for which a more robust methodology should be considered in order to obtain more realistic exposures and conclude on potential health risks.

## Figures and Tables

**Table 1:** Mean exposures estimates (mg kg BW^−1^ day^−1^) for food colors listed in the Brazilian legislation and for which numerical ADIs are set by the JECFA.

INS	Food Color	ADI (mg kg BW^−1^ day)	Exposure (% ADI) POF 2008–2009	Exposure (% ADI) POF 2017–2018	% Exposure Variation (POF 2017–2018 in Relation to POF 2008–2009)
100(i)	Curcumin	0–3 ^1^	1.6 (54)	1.2 (41)	−25
102	Tartrazine	0–10 ^2^	0.4 (4)	0.3 (3)	−25
104	Quinoline yellow	0–3 ^2^	0.2 (6)	0.2 (6)	0
110	Sunset yellow FCF	0–4 ^3^	0.5 (12)	0.3 (8)	−40
120	Carmines	0–5 ^1^	1.5 (30)	1.1 (22)	−27
122	Azorubine	0–4 ^5^	0.2 (6)	0.2 (4)	0
123	Amaranth	0–0.5 ^6,7^	0.1 (25)	0.1 (20)	0
124	Ponceau 4R	0–4 ^3,8^	0.5 (13)	0.3 (7)	−40
127	Erythrosine	0–0.1 ^1,6^	0.02 (23)	0.02 (21)	0
129	Allura red AC	0–7 ^2^	0.6 (8)	0.4 (6)	−33
132	Indigotin	0–5 ^9^	0.6 (11)	0.4 (8)	−33
133	Brilliant blue FCF	0–6 ^1^	0.7 (12)	0.3 (6)	−57
141(i)	Chlorophylls, copper complexes	0–15 ^10^	1.7 (12)	1.1 (7)	−35
141(ii)	Chlorophyllin copper complexes, potassium, and sodium salts	0–15 ^11^	1.4 (9)	0.8 (6)	−43
143	Fast green FCF	0–25 ^12^	0.3 (1)	0.2 (1)	−33
150b	Caramel II—sulfite caramel	0–160 ^l2^	180.7 (113)	151.3 (95)	−16
150c	Caramel III—ammonia caramel	0–150 ^l2^	145.3 (97)	111.5 (74)	−23
150d	Caramel IV—sulfite ammonia caramel	0–150 ^l2^	204.7 (136)	171.5 (114)	−16
151	Brilliant black	0–1 ^13^	0.2 (19)	0.2 (19)	0
155	Brown HT	0–1.5 ^7^	0.1 (7)	0.1 (7)	0
160a(i)	Carotenes, beta-, synthetic	0.1 ^13,14^	0.5–1.0 (510–1009)	0.4–0.6 (414–591)	−20 to −40
160a(iii)	Carotenes, beta-, *Blakeslea trispora*				
160b(i)	Annatto extracts, bixin-based	0–12 ^15^	0.5 (4)	0.3 (3)	−40
160b(ii)	Annatto extracts, norbixin-based	0–0.6 ^15^	0.5 (79)	0.3 (52)	−40
160c(ii)	Paprika extract	0–1.5 ^1^	1.2 (80)	0.8 (56)	−33
160e	Carotenal, beta-apo-8′-	0–0.3 ^14^	0.5 (166)	0.4 (132)	−20
161g	Canthaxanthin	0–0.3 ^14^	0.04 (14)	0.04 (13)	0
163(ii)	Grape skin extract	0–2.5 ^4^	0.2 (8)	0.2 (6)	0
163(iii)	Blackcurrant extract	0–2.5 ^1,4,16,17^	5.8 (232)	4.8 (194)	−17
172(i), (ii), (iii)	Iron oxide (black, red, yellow)	0–0.5 ^18^	0.2 (34)	0.1 (15)	−50
183	Jagua (genipin–glycine) blue	0–11 ^19^	0.02 (0.2)	0.01 (0,1)	−50

^1^ Ref. [24]. ^2^ Ref. [25]. ^3^ Ref. [26]. ^4^ MLs in the legislation do not specify if they are expressed as anthocyanins, but the JECFA ADI and *Codex Alimentarius* levels in the General Standard for Food Additives (GSFA) [1] are expressed as anthocyanins. In the case of the grape skin extract, it was considered that it contained 3% anthocyanins as detailed in the JECFA evaluations, but the levels of anthocyanins in the blackcurrant extract were not found [27]. ^5^ Ref. [28]. ^6^ Ref. [29]. ^7^ Ref. [30]. ^8^ Ref. [31]. ^9^ Ref. [32]. ^10^ Ref. [33]. ^11^ Ref. [34]. ^12^ Ref. [35]. ^13^ Ref. [36]. ^14^ As no ADI is set for beta-carotenes by the JECFA, it was considered that 6 mg.day^−1^ was not of concern for a 60 kg adult, based on the evaluation of the Committee [36]. ^15^ Ref. [37]. ^16^ Ref. [38]. ^17^ Although no safety level is identified by the JECFA for the blackcurrant extract [24,27,38], the European Food Safety Authority (EFSA) evaluated anthocyanins and considered an ADI of 0–2.5 mg kg BW^−1^ day^−1^ for both grape skin and blackcurrant extracts [39]. ^18^ Ref. [40]. ^19^ Ref. [41].

**Table 2:** High-exposure (P90 and P95) estimates (mg kg BW^−1^ day^−1^) for food colors permitted in the Brazilian legislation.

INS	Exposure P90 (% ADI)			Exposure P95 (% ADI)		
	POF 2008–2009	POF 2017–2018	% Variation (POF 2017–2018 in Relation to POF 2008–2009)	POF 2008–2009	POF 2017–2018	% Variation (POF 2017–2018 in Relation to POF 2008–2009)
100(i)	2.4 (82)	1.8 (62)	−33	2.8 (95)	2.1 (72)	−25
102	1.1 (11)	1.1 (11)	0	1.4 (14)	1.5 (15)	7
104	0.9 (31)	1.0 (32)	11	1.2 (39)	1.3 (45)	8
110	2.7 (67)	1.1 (27)	−59	3.3 (82)	1.5 (37)	−55
120	2.7 (54)	2.0 (40)	−26	3.1 (62)	2.4 (47)	−22
122	0.6 (15)	0.6 (14)	0	0.7 (18)	0.8 (19)	14
123	0.5 (100)	0.5 (100)	0	0.6 (124)	0.7 (135)	17
124	4.2 (105)	1.9 (48)	−55	5.2 (130)	3.1 (77)	−40
127	0.10 (100)	0.10 (100)	0	0.12 (123)	0.14 (136)	17
129	2.8 (40)	1.2 (17)	−57	3.4 (48)	1.5 (22)	−56
132	2.8 (55)	1.2 (23)	−57	3.4 (67)	1.5 (31)	−56
133	4.4 (73)	1.1 (18)	−75	5.4 (89)	1.5 (25)	−72
141(i)	3.9 (26)	3.3 (22)	−18	4.7 (31)	4.5 (30)	−4
141(ii)	3.6 (24)	3.1 (21)	−14	4.3 (29)	4.3 (29)	0
143	0.7 (3)	0.6 (2)	−14	0.8 (3)	0.8 (3)	0
150b	549.9 (344)	533.8 (334)	−3	677.4 (423)	726.5 (454)	7
150c	310.7 (207)	250.6 (167)	−24	360.7 (240)	305.2 (203)	−15
150d	573.8 (383)	553.9 (369)	−3	701.4 (468)	746.6 (498)	6
151	0.9 (93)	1.0 (95)	11	1.2 (118)	1.3 (134)	8
155	0.5 (32)	0.5 (33)	0	0.6 (40)	0.7 (46)	17
160a(i)	2.0–4.1 (1951–4075)	0.7–2.9 (672–2916)	−41 to −65	2.4–5.1 (2351–5075)	0.8–3.8 (789–3763)	−25 to −67
160a(iii)						
160b(i)	1.6 (13)	0.7 (6)	−56	1.9 (15)	0.9 (7)	−53
160b(ii)	1.6 (259)	0.7 (116)	−56	1.9 (309)	0.9 (143)	−53
160c(ii)	2.0 (135)	1.5 (100)	−25	2.4 (162)	1.8 (117)	−25
160e	1.2 (412)	1.2 (387)	0	1.5 (497)	1.5 (515)	0
161g	0.26 (86)	0.12 (39)	−54	0.32 (106)	0.15 (51)	−53
163(ii)	0.7 (27)	0.6 (23)	−14	0.8 (33)	0.7 (29)	0
163(iii)	22.3 (894)	18.7 (750)	−16	27.3 (1094)	24.2 (968)	−11
172(i), (ii), (iii)	2.0 (401)	0.9 (181)	−55	2.5 (501)	1.5 (294)	−40
183	0.37 (3)	0.30 (2,7)	−19	0.55 (5)	(3,4)	−33

**Table 3:** Food categories that contributed more than 5% to the mean exposure estimates for each food color.

Food Categories	POF 2008–2009 (% Contribution)	POF 2017–2018 (% Contribution)
Water-based flavored beverages	Quinoline yellow (87%), Brilliant black (84%), Brown HT (74%), Erythrosine (69%), Amaranth (65%), Caramel II (sulfite caramel) (45%), Tartrazine (43%), Caramel IV (sulfite ammonia caramel) (39%), Azorubine (36%), Chlorophyllin copper complexes, potassium, and sodium salts (36%), Sunset yellow FCF (33%), Carotenal, beta-apo-8′ (32%), Indigotin (29%), Allura red AC (29%), Chlorophylls, copper complexes (28%), Fast green FCF (28%), Brilliant blue FCF (23%), Annatto extracts, norbixin-based (17%), Annatto extracts, bixin-based (17%), Ponceau 4R (16%), Carmines (12%), Curcumin (10%), Grape skin extract (8%), Blackcurrant extract (8%), Carotenes, beta-, synthetic (8%), Caramel III (ammonia caramel) (6%)	Quinoline yellow (82%), Brilliant black (81%), Amaranth (77%), Erythrosine (74%), Brown HT (68%), Chlorophyllin copper complexes, potassium, and sodium salts (56%), Caramel II (sulfite caramel) (51%), Tartrazine (50%), Sunset yellow FCF (47%), Caramel IV (sulfite ammonia caramel) (45%), Chlorophylls, copper complexes (44%), Azorubine (43%), Indigotin (40%), Carotenal, beta-apo-8′ (39%), Allura red AC (39%), Brilliant blue FCF (35%), Fast green FCF (35%), Ponceau 4R (27%), Annatto extracts, norbixin-based (25%), Annatto extracts, bixin-based (25%), Carmines (14%), Curcumin (13%), Blackcurrant extract (10%), Grape skin extract (9%), Carotenes, beta-, synthetic (9%), Caramel III (ammonia caramel) (7%), Paprika extract (6%)
Soups and broths	Canthaxanthin (55%), Carotenal, beta-apo-8′ (32%), Carotenes, beta-, synthetic (31%), Annatto extracts, norbixin-based (25%), Annatto extracts, bixin-based (25%), Chlorophyllin copper complexes, potassium, and sodium salts (23%), Azorubine (18%), Chlorophylls, copper complexes (18%), Caramel III (ammonia caramel) (14%), Caramel II (sulfite caramel) (11%), Tartrazine (11%), Caramel IV (sulfite ammonia caramel) (10%), Paprika extract (10%), Sunset yellow FCF (8%), Ponceau 4R (8%), Indigotin (7%), Allura red AC (7%), Blackcurrant extract (7%), Grape skin extract (6%), Brilliant blue FCF (6%)	-
Fish and fish products, including mollusks, crustaceans, and echinoderms	Iron oxides (96%), Ponceau 4R (62%), Brilliant blue FCF (47%), Sunset yellow FCF (40%), Indigotin (35%), Allura red AC (34%), Chlorophyllin copper complexes, potassium, and sodium salts (24%), Fast green FCF (23%), Canthaxanthin (22%), Carmines (22%), Chlorophylls, copper complexes (19%), Amaranth (16%), Carotenal, beta-apo-8′ (13%), Carotenes, beta-, synthetic (13%), Caramel III (ammonia caramel) (13%), Grape skin extract (10%), Caramel IV (sulfite ammonia caramel) (9%), Carotenes, beta-, Blakeslea trispora (6%)	Iron oxides (84%), Ponceau 4R (45%), Brilliant blue FCF (29%), Sunset yellow FCF (24%), Indigotin (20%), Allura red AC (20%), Chlorophyllin copper complexes, potassium, and sodium salts (15%), Carmines (12%), Chlorophylls, copper complexes (12%), Fast green FCF (12%), Canthaxanthin (10%), Amaranth (8%), Caramel III (ammonia caramel) (7%), Carotenal, beta-apo-8′ (6%), Carotenes, beta-, synthetic (6%), Grape skin extract (5%)
Pasta and noodles	Blackcurrant extract (60%), Grape skin extract (54%), Caramel III (ammonia caramel) (24%), Curcumin (21%), Caramel II (sulfite caramel) (19%), Caramel IV (sulfite ammonia caramel) (17%), Annatto extracts, norbixin-based (15%), Annatto extracts, bixin-based (15%), Paprika extract (9%), Carotenal, beta-apo-8′ (6%), Carotenes, beta-, synthetic (5%)	Blackcurrant extract (57%), Grape skin extract (55%), Caramel III (ammonia caramel) (25%), Curcumin (23%), Annatto extracts, norbixin-based (18%), Annatto extracts, bixin-based (18%), Caramel II (sulfite caramel) (16%), Caramel IV (sulfite ammonia caramel) (16%), Paprika extract (10%), Chlorophyllin copper complexes, potassium, and sodium salts (7%), Carotenal, beta-apo-8′ (6%), Carotenes, beta-, synthetic (5%), Carmines (5%), Chlorophylls, copper complexes (5%)
Prepared foods	Blackcurrant extract (12%), Grape skin extract (11%), Canthaxanthin (10%), Carotenal, beta-apo-8′ (6%), Carotenes, beta-, synthetic (5%)	Blackcurrant extract (23%), Grape skin extract (22%), Canthaxanthin (17%), Carotenal, beta-apo-8′ (11%), Chlorophyllin copper complexes, potassium, and sodium salts (11%), Annatto extracts, norbixin-based (11%), Carotenes, beta-, synthetic (11%), Annatto extracts, bixin-based (11%), Caramel III (ammonia caramel) (10%), Chlorophylls, copper complexes (9%), Caramel II (sulfite caramel) (7%), Caramel IV (sulfite ammonia caramel) (7%), Azorubine (6%)
Crackers	Fast green FCF (16%), Tartrazine (13%), Carotenes, beta-, synthetic (9%), Caramel III (ammonia caramel) (8%), Indigotin (8%), Allura red AC (8%), Brilliant blue FCF (7%), Caramel II (sulfite caramel) (6%), Caramel IV (sulfite ammonia caramel) (6%)	Fast green FCF (21%), Tartrazine (15%), Indigotin (12%), Allura red AC (12%), Carotenes, beta-, synthetic (11%), Brilliant blue FCF (10%), Caramel III (ammonia caramel) (10%), Caramel II (sulfite caramel) (7%), Caramel IV (sulfite ammonia caramel) (7%), Azorubine (6%)
Meat and meat products, including poultry and game	Carmines (14%), Annatto extracts, norbixin-based (9%), Annatto extracts, bixin-based (9%), Caramel III (ammonia caramel) (7%), Caramel II (sulfite caramel) (6%), Paprika extract (6%), Caramel IV (sulfite ammonia caramel) (5%)	Carmines (18%), Annatto extracts, norbixin-based (13%), Annatto extracts, bixin-based (13%), Caramel III (ammonia caramel) (9%), Paprika extract (8%), Caramel II (sulfite caramel) (7%), Caramel IV (sulfite ammonia caramel) (6%)
Fruit juices and nectars	Carotenes, beta-, *Blakeslea trispora* (93%), Carmines (25%), Chlorophylls, copper complexes (22%), Annatto extracts, bixin-based (20%), Annatto extracts, norbixin-based (20%), Blackcurrant extract (10%), Grape skin extract (9%)	Carotenes, beta-, *Blakeslea trispora* (95%), Carmines (21%), Chlorophylls, copper complexes (21%), Annatto extracts, norbixin-based (18%), Annatto extracts, bixin-based (18%), Grape skin extract (7%), Blackcurrant extract (7%)
Fine bakery wares (sweet, salty, and savory) and mixes	Fast green FCF (18%), Tartrazine (14%), Caramel III (ammonia caramel) (9%), Indigotin (9%), Allura red AC (9%), Brilliant blue FCF (7%), Caramel II (sulfite caramel) (7%), Azorubine (6%)	Fast green FCF (17%), Tartrazine (12%), Indigotin (9%), Allura red AC (9%), Brilliant blue FCF (8%), Caramel III (ammonia caramel) (8%), Caramel II (sulfite caramel) (6%), Azorubine (5%)
Fermented milks	Carmines (11%)	Carmines (10%)
Edible ices, including sherbet and sorbet	Brown HT (11%), Amaranth (7%), Quinoline yellow (7%), Brilliant black (6%)	Brown HT (6%)
Fruit preparations, including pulp, purees, and fruit toppings	Jagua (genipin–glycine) blue (68%), Erythrosine (20%), Azorubine (11%)	Jagua (genipin–glycine) blue (81%), Erythrosine (16%), Azorubine (9%)
Beer	Caramel III (ammonia caramel) (12%), Caramel IV (sulfite ammonia caramel) (9%)	Caramel III (ammonia caramel) (20%), Caramel IV (sulfite ammonia caramel) (13%)
Fat spreads, dairy fat spreads, and blended spreads	Canthaxanthin (11%)	Canthaxanthin (69%), Carotenal, beta-apo-8′ (23%), Carotenes, beta-, synthetic (22%)
Snacks—potato-, cereal-, flour-, or starch-based (from roots and tubers, pulses, and legumes)	Brown HT (8%)	Brown HT (15%), Azorubine (9%), Brilliant black (9%), Quinoline yellow (8%), Sunset yellow FCF (5%), Tartrazine (5%)
Cheese	Carotenes, beta-, synthetic (9%)	Carotenes, beta-, synthetic (12%)
Peeled, cut, or shredded fresh vegetables (including mushrooms and fungi)	Paprika extract (65%), Curcumin (48%)	Paprika extract (66%), Curcumin (45%)
Food supplements	-	Iron oxides (14%)
Jams and jellies	-	Jagua (genipin–glycine) blue (6%)
Flavored fluid milk drinks	-	Azorubine (6%)
Non-emulsified sauces	-	Brown HT (7%)
Soybean-based beverages	Jagua (genipin–glycine) blue (27%)	Jagua (genipin–glycine) blue (11%)

The trace “-“ indicates that no food color had an intake that contributed more than 5%.

**Table 4:** The classification of food colors based on the exceedance of the acceptable daily intake (ADI). Low priority: mean and high estimates below the ADI; medium priority: mean estimates below the ADI and high estimates near or above the ADI; high priority: mean and high estimates above the ADI.

Low Priority	Medium Priority	High Priority
Curcumin	Canthaxanthin	Caramel II
Carmines	Amaranth	Caramel IV
Quinoline yellow	Brilliant black	Synthetic beta-carotenes
Grape skin extract	Erythrosine	Beta-carotenes from *B. trispora*
Indigotin	Ponceau 4R	Beta-apo-8′-carotenal
Brown HT	Iron oxide black	Blackcurrant extract
Sunset yellow FCF	Iron oxide red	
Chlorophyllin copper complexes, potassium, and sodium salts	Iron oxide yellow	
Brilliant blue FCF	Annatto extracts, norbixin-based	
Allura red AC	Paprika extract	
Azorubine	Caramel III	
Chlorophylls, copper complexes		
Tartrazine		
Annatto extracts, bixin-based		
Fast green FCF		
Jagua (genipin–glycine) blue		

## Data Availability

The original contributions presented in the study are included in the article, further inquiries can be directed to the corresponding author.
